# The TLR2 is activated by sporozoites and suppresses intrahepatic rodent malaria parasite development

**DOI:** 10.1038/srep18239

**Published:** 2015-12-15

**Authors:** Hong Zheng, Zhangping Tan, TaoLi Zhou, Feng Zhu, Yan Ding, Taiping Liu, Yuzhang Wu, Wenyue Xu

**Affiliations:** 1 Department of Pathogenic Biology, Third Military Medical University, Chongqing, 400038, P. R. China; 2 Institute of Immunology of PLA, Third Military Medical University, Chongqing, 400038, P. R. China

## Abstract

TLRs (Toll-like receptors) play an important role in the initiation of innate immune responses against invading microorganisms. Although several TLRs have been reported to be involved in the innate immune response against the blood-stage of malaria parasites, the role of TLRs in the development of the pre-erythrocytic stage is still largely unknown. Here, we found that sporozoite and its lysate could significantly activate the TLR2, and induce macrophages to release proinflammatory cytokines, including IL-6, MCP-1 and TNF-α, in a TLR2-dependent manner. Further studies showed that sporozoite and its lysate could be recognized by either TLR2 homodimers or TLR2/1 and TLR2/6 heterodimers, implicating the complexity of TLR2 agonist in sporozoite. Interestingly, the TLR2 signaling can significantly suppress the development of the pre-erythrocytic stage of *Plasmodium yoelii*, as both liver parasite load and subsequent parasitemia were significantly elevated in both TLR2- and MyD88-deficient mice. Additionally, the observed higher level of parasite burden in TLR2^−/−^ mice was found to be closely associated with a reduction in proinflammatory cytokines in the liver. Therefore, we provide the first evidence that sporozoites can activate the TLR2 signaling, which in turn significantly inhibits the intrahepatic parasites. This may provide us with novel clues to design preventive anti-malaria therapies.

Malaria is still one of the most devastating diseases worldwide. Approximately 40% of the world’s population is at risk for malaria, and 200 million new cases with 650,000 deaths occur each year^1^. Malaria begins with the bite of Plasmodium-infected mosquitoes. After being injected into the host’s skin, sporozoites rapidly invade the liver and initiate the intrahepatic development; then, the matured schizonts released from hepatocytes invade the red blood cells and initiate the development of the blood stage, which causes the clinical symptoms. In contrast to the blood stage, the pre-erythrocytic stage does not lead to any clinical symptoms. Thus, understanding the mechanism of host immune responses against the pre-erythrocytic stage of malaria parasite could provide us with clues to design prophylactic strategies.

Although great progress has been made to elucidate the mechanisms of protective immunity against the pre-erythrocytic stage induced by irradiation- or genetically attenuated sporozoites^2^,^3^,^4^, relatively little is known about the protective immune response against the malaria parasite during primary infection. It has been reported that both liver NK cells, CD49b^+^ CD3^+^ NKT and CD4^−^ CD8^−^ NK1.1^+^ TCRαβ intermediate cells (DN NKT cells) could be activated by sporozoite challenge and suppress the development of the malaria pre-erythrocytic stage^5^,^6^,^7^. γδ T cells have also been found to exhibit inhibitory effects on intrahepatic parasites^8^. It appeared that innate immune responses could play an inhibitory role in the development of the pre-erythrocytic stage of malaria parasite. However, to our knowledge, it is still largely unknown how the sporozoites trigger innate immune responses.

TLRs (Toll-like receptors) are the main PRRs (pattern recognition receptors) on myeloid cells and have an important role in detecting a variety of invading microorganisms and eliciting early innate immune responses. Mice express 12 TLRs mediating cellular recognition of different PAMPs (pathogen associated molecular patterns)^9^. While TLR9 mediates the recognition of the CpG motif in bacterial DNA, TLR4 mediates the recognition of the LPS (lipopolysaccharide) of Gram-negative bacteria, and TLR2 was involved in the recognition of a wide range of ligands through heterodimerization with TLR1 or TLR6, including lipopeptides^10^, peptidoglycan^11^, lipoteichoic acid^12^, lipoarabinomannan^13^ and zymosan^14^. In the case of the malaria parasite, glycosylphosphatidylinositols (GPIs) of *Plasmodium falciparum* blood-stage were recognized by TLR2 and to some extent by TLR4 and were critical for the macrophages to generate proinflammatory cytokines^15^,^16^. Hemozoin or hemozoin/malarial DNA could activate TLR9^17^,^18^, which was recently demonstrated to be essential for controlling the growth of the malaria blood stage^19^.

Therefore, we hypothesized that TLRs might also be involved in the recognition of invading sporozoites and initiate innate immune responses against intrahepatic parasites. Our previous study showed that pre-treatment with individual TLR agonists could variously inhibit intrahepatic parasites through the upregulation of proinflammatory cytokines^20^,^21^. However, it is not known which TLRs are actually involved in the recognition of invading sporozoites and the suppression of pre-erythrocytic stage development during primary infection. In this report, we found that TLR2 was involved in the recognition of sporozoites and induced peritoneal macrophages to release proinflammatory cytokines. Then, we also investigated its influence on the development of the pre-erythrocytic stage *in vivo*, and the underlying mechanism.

## Results

### P. yoelii sporozoite lysate significantly activates TLR2

**Figure 1:**
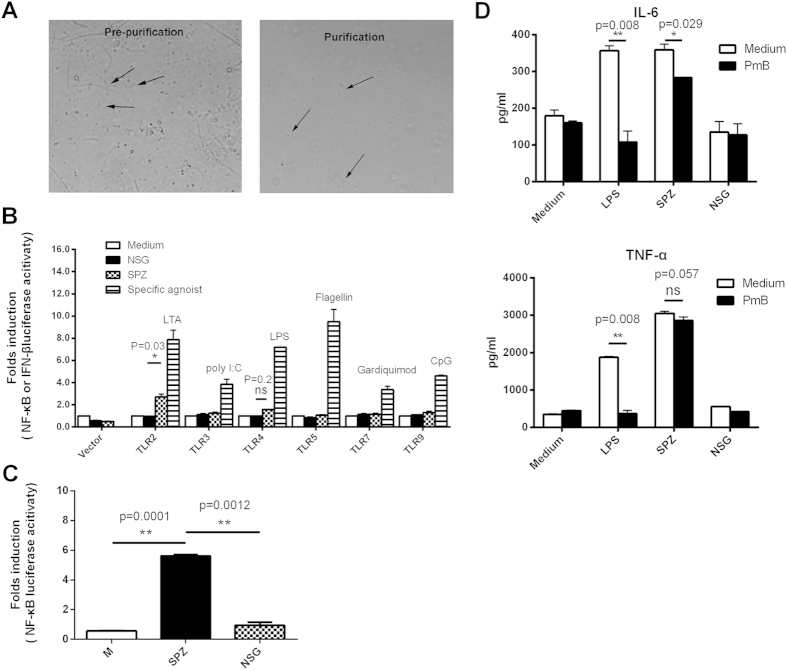
The purified sporozoites and their TLRs activities. (**A**), Microscopy of the contamination in the sporozoites before or after purification by DEAE-cellulose chromatography. (**B**), After HEK 293FT cells/well were plated in 96-well plate for 24 h, each well was transfected with the indicated TLRs along with TK-RL and pBIIx-luc or pGL3-IFN-β. 24 h later, cells were stimulated with the indicated positive TLR agonist, SPZ lysate or NSG lysate. After incubation for 6 h or 18 h, either NF-κB (TLR2, TLR4, TLR5, TLR7 and TLR9) or IFN-β gene (TLR3) reporter activity was determined. Both NF-κB and IFN-β gene reporter activity were expressed as the ratio of Renilla luciferase activity to firefly luciferase activity. (**C**), TLR2-transfected HEK293FT cells were stimulated with whole sporozoites lack of infectivity (cell/sporozoite = 1:5) for 6 h, then the NF-κB gene reporter activity was then determined. (**D**), Peritoneal macrophages were stimulated with LPS, SPZ or NSG treated with or without PmB, and the concentrations of both TNF-α and IL-6 in the supernatants were determined using CBA beads. All the experiments were repeated three times, and all data were presented as the mean ± SD, *p < 0.05; **p < 0.01.

In contrast to the blood stage, it is still unknown which TLRs are involved in the recognition of sporozoites, possibly due to the difficulty in obtaining enough high-purified sporozoites. To obtain sporozoite with high purity, we soaked mosquitoes in 75% ethanol to remove the surface contamination, and the sporozoites released from the salivary gland were purified using DEAE-cellulose chromatography, which could efficiently remove endotoxin^22^. Few contaminants were found in the purified sporozoite under light microscope (Fig. 1A). The endotoxin in the purified SPZ (sporozoite) lysate was only about 1 ng/ml, which was much lower than what had been previously reported (30 ng/ml)^23^. The bacteria plating also showed only about one bacterial colony per sporozoite remaining in the purified SPZ lysate (data not shown). Therefore, most contaminants were efficiently removed during our preparation of SPZ lysate.

To test whether known TLRs were involved in the recognition of sporozoites, either NF-κB or IFN-β reporter gene activity in individual TLR-transfected HEK293T cells was determined following stimulation with SPZ or NSG lysate. As shown in Fig. 1B, no signal was detected in any TLR-transfected HEK293T cells when stimulated with NSG lysate, further confirming the effective removal of the contaminants during the purification process. Although each commercial positive agonist, such as TLR2 agonist LTA-BS, TLR3 agonist poly I:C, TLR4 agonist LPS, TLR5 agonist Flagellin, TLR7 agonist Gardiquimod^TM^ and TLR9 agonist CpG ODN, could greatly activate the NF-κB reporter gene in TLR2, TLR4 or TLR5, TLR7 or TLR9-transfected cells and the IFN-β reporter gene in TLR3-transfected cells, SPZ lysate could only significantly activate TLR2-transfected cells, with over 2-fold of NF-κB reporter gene activity of the baseline (*P < 0.05*). To investigate whether the activation of TLR2 by SPZ lysate is physiological relevance, the whole sporozoite lack of infectivity was prepared by placing in column buffer at 4 °C for more than 24 h as previously described^24^,^25^, and then stimulated the TLR2-transfected HEK293FT cells. As same as the lysate, the whole sporozoites could also induce more than 4 folds of NF-κB reporter activity as compared to the control (Fig. 1C).

However, a slight increase in NF-κB reporter gene activity was also observed in TLR4-transfected cells following stimulation with SPZ lysate (*P > 0.05*). To test whether the observed TLR4 activation was resulted from SPZ or its contaminating endotoxin, the effect of PmB (Polymyxin B) treatment on the production of proinflammatory cytokines in peritoneal macrophages stimulated with LPS or SPZ lysate was then investigated. As shown in Fig. 1D, the levels of both TNF-α and IL-6 released by LPS-stimulated macrophages were reduced to baseline after treatment with PmB, indicating the efficient competitive binding of PmB to LPS. Although the level of TNF-α released by macrophages was comparable when stimulated with SPZ lysate treated with or without PmB (*P > 0.05*), the level of IL-6 released by SPZ-stimulated macrophages was significantly reduced after treatment with PmB (*P < 0.05*). These data suggested that the observed slight TLR4 activity might be attributed to LPS contamination in SPZ.

### Characterization of the TLR2 agonist contained in P. yoelii sporozoites

**Figure 2:**
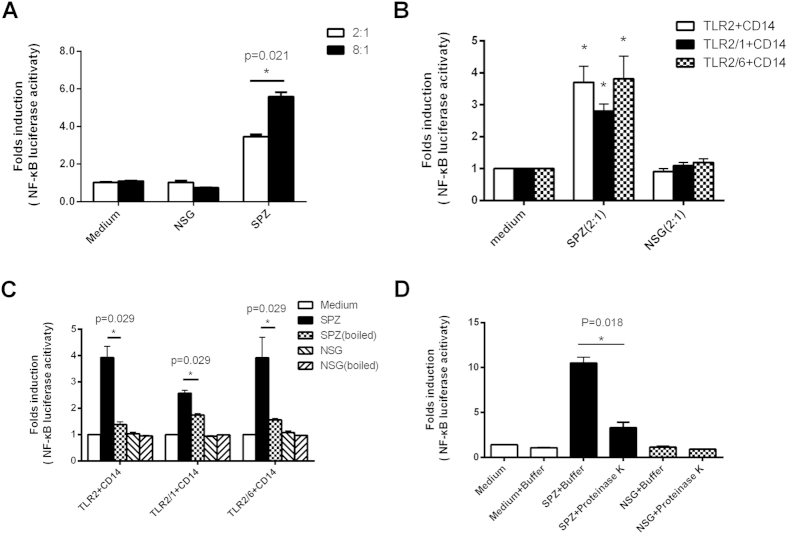
Characterization of TLR2 agonist contained in sporozoites. (**A**), TLR2 -transfected cells were stimulated with the increased concentration of SPZ or NSG lysate, and the NF-κB gene reporter activity was then determined. (**B**), TLR2/CD14-, TLR2/1/CD14- or TLR2/6/CD14-transfected cells were stimulated with the indicated concentration of SPZ lysate or NSG lysate, and NF-κB gene reporter activity was determined. (**C**), TLR2/CD14-, TLR2/1/CD14- or TLR2/6/CD14-transfected cells were stimulated with boiled or unboiled SPZ or NSG lysate, and then NF-κB gene reporter activity was determined. (**D**), TLR2 -transfected cells (cell/sporozoites = 1:10)were stimulated with the SPZ or NSG lysate pretreated with or without protease K, and the NF-κB gene reporter activity was then determined. The NF-κB gene reporter activity was expressed as the ratio of Renilla luciferase activity to firefly luciferase activity. All the experiments were repeated three times, and all data were presented as the mean ± SD, *p < 0.05.

The NF-κB reporter activity of TLR2-transfected HEK293FT significantly augmented when the dose of whole SPZ and its lysate, but not NSG lysate, increased from 2:1 to 8:1(SPZ:cell, *P < 0.05*, Fig. S1& Fig. 2A), implicating that whole SPZ and its lysate could activate TLR2 in a dose-dependent manner. In addition to homodimer, TLR2 can also exist as a heterodimer with TLR1 or TLR6 and specifically recognize wider range of ligands^26^. To test whether TLR2 agonist in SPZ could activate either TLR2 homodimer or heterodimer, NF-κB reporter activity was assessed after the TLR2/2, or TLR2/1 or TLR2/6-transfected HEK293FT was stimulated with SPZ lysate, respectively. As shown in Fig. 2B, SPZ lysate, but not NSG lysate, could also induce high levels of NF-κB reporter activity through either TLR2 homodimers or TLR2 heterodimers with TLR1 or TLR6, indicating the complexity of the TLR2 agonists contained in sporozoites.

As TLR2 agonist could be some protein^27^,^28^, we then stimulated TLR2/2, or TLR2/1 or TLR2/6-transfected HEK293FT with SPZ lysate boiled or not. Interestingly, the NF-κB reporter activity of TLR2/2, TLR2/1 and TLR2/6-transfected HEK293FT cells was greatly reduced when the sporozoite lysate was heat-inactivated (Fig. 2C). The activity of TLR2-transfected HEK293FT cells was also greatly reduced when stimulated with SPZ lysate treated with proteinase K (Fig. 2D). To confirm this finding is physiological relevance, we also used the whole sporozoite to repeat the experiments, and similar results were obtained (Fig. S2). Thus, our data strongly suggest that the protein of sporozoite might be involved in activating TLR2.

### P. yoelii sporozoite lysate induces peritoneal macrophages to release pro-inflammatory cytokines in a TLR2-dependent manner

**Figure 3:**
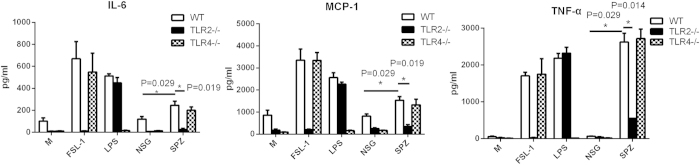
*P. yoelii* sporozoite lysate induced peritoneal macrophages to release proinflammatory cytokines in a TLR2-dependent manner. Peritoneal macrophages collected from WT, TLR2^−/−^ or TLR4^−/−^ mice were stimulated with or without SPZ lysate, FSL-1, LPS or NSG lysate. 17 h later, the concentration of IL-6, MCP-1 and TNF-α in the supernatant was determined using CAB beads. The experiment was repeated three times, and all data were presented as the mean ± SD, *p < 0.05.

To confirm whether sporozoites could specifically activate TLR2, proinflammatory cytokines, such as IL-6, MCP-1 and TNF-α, were detected in peritoneal macrophages from WT, TLR2^−/−^ and TLR4^−/−^ mice following stimulation with SPZ lysate. As shown in Fig. 3, a little bit more IL-6, MCP-1 or TNF-α was released from WT than either TLR4^−/−^ or TLR2^−/−^ mouse peritoneal macrophages without stimulus, but the levels of proinflammatory cytokines produced by either WT, TLR2^−/−^ or TLR4^−/−^ mouse macrophages were comparable when stimulated with NSG lysate or incubated with medium. It seems that a low level of TLR2 and TLR4 agonists were contaminated in medium, but not specific for the NSG. In contrast, approximately 2-fold higher of both IL-6 and MCP-1 were produced by WT mouse peritoneal macrophages stimulated with SPZ lysate than stimulated with NSG lysate (*P < 0.05*). The level of TNF-α was much higher in SPZ lysate-induced peritoneal macrophages than that in NSG lysate-induced peritoneal macrophages (*P < 0.01*). However, the level of all three proinflammatory cytokines released by TLR2^−/−^ macrophages was almost at baseline when incubated with SPZ lysate or TLR2 agonist FSL-1. Additionally, the level of either IL-6, TNF-α or MCP-1 was also greatly reduced when the TLR4^−/−^ macrophages were stimulated with LPS. To verify this finding, we also used the whole sporozoite to stimulate the peritoneal macrophages from the naïve, TLR2^−/−^ and TLR4^−/−^ mice, and similar results were obtained (Fig. S3).Thus, these data demonstrated that SPZ and its lysate could specifically activate macrophages in a TLR2-dependent manner.

### TLR2 suppresses the development of the P. yoelii pre-erythrocytic stage

**Figure 4:**
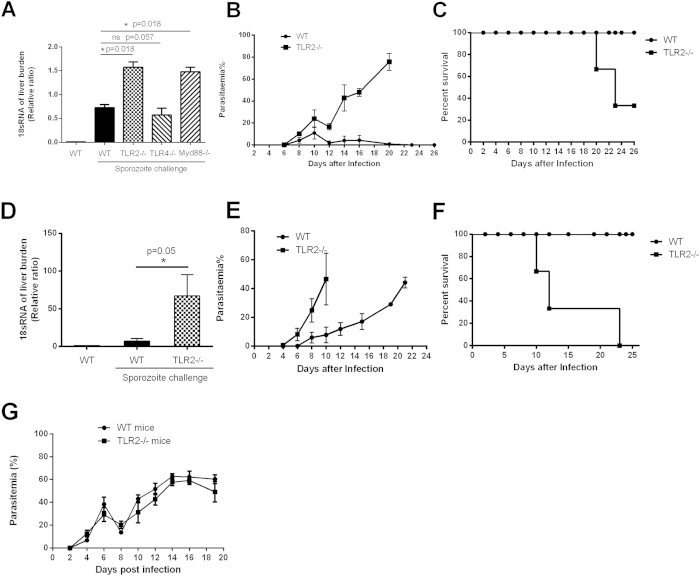
TLR2 deficiency promotes the growth of *plasmodium yoelii*. (**A–C**), WT, MyD88^−/−^, TLR4^−/−^ and TLR2^−/−^ mice (n = 6) were challenged intravenously with sporozoites. 42 h later, liver parasite burden (**A**) was detected by quantitative PCR, parasitemia was examined by Giemsa staining (**B**), and survival rate (**C**) was recorded. D–F, WT and TLR2^−/−^ mice (n = 6) were challenged through the bite of infected mosquitoes. 42 h later, liver parasite burden (**D**) was detected by quantitative PCR, and parasitemia (**E**) and survival rate (**F**) were recorded. G, WT and TLR2^−/−^ mice (n = 6) were injected intraperitoneally with the parasitized RBC, and the parasitemia was determined every two days. All the experiments were repeated for three times, and all the data were presented as the mean ± SD, *p < 0.05.

Next, we investigated the role of TLR2 in the development of the pre-erythrocytic stage. Both liver parasite burden and blood parasitemia were compared among WT, TLR2-, TLR4- and MyD88-deficient mice following intravenous *P. yoelii* sporozoite challenge. Although there was no significant difference of liver parasite load between TLR4^−/−^ and WT mice, the liver parasite burden of TLR2- or adaptor MyD88-deficient mice was about 2-fold higher than that of WT mice (Fig. 4A, *p < 0.05*). Consistent with the liver parasite burden, the level of parasitemia in TLR2-deficient mice was also significantly higher than that of WT mice during the course of infection. Malaria parasite growth was uncontrolled from day 12 in TLR2-deficient mice, and parasitemia could reach at peak of 70% at day 20. However, parasites were almost cleared in all WT mice by day 23 (Fig. 4B,C).

It is well known that mosquito bite is the natural infection route of malaria parasites. To further confirm the role of the TLR2 signaling in inhibiting the development of the pre-erythrocytic stage, both liver parasite burden and parasitemia were compared in WT and TLR2^−/−^ mice following sporozoite challenge by malaria parasite-infected mosquito bite. Liver parasite burden in TLR2^−/−^ mice was much higher than that of WT mice (*p < 0.05*), and parasites in the peripheral blood appeared 2 days earlier in TLR2^−/−^ mice than in WT mice. In addition, the malaria parasites in TLR2^−/−^ mice grew rapidly, and over 60% of mice died by day 12, while all the malaria parasites were cleared from the WT mice (Fig. 4D–F).

The high parasitemia of *P. yoelii* in TLR2^−/−^ mice was resulted from their higher liver parasite burden, because TLR2 deficiency could not significantly promote the development of the blood stage (Fig. 4G), which is consistent with the previous study^19^. Therefore, our data strongly support that TLR2 signaling could significantly inhibit the development of the malaria pre-erythrocytic stage.

### Higher levels of liver parasite burden in TLR2-deficient mice were closely associated with the reduced production of proinflammatory cytokines in liver

**Figure 5:**
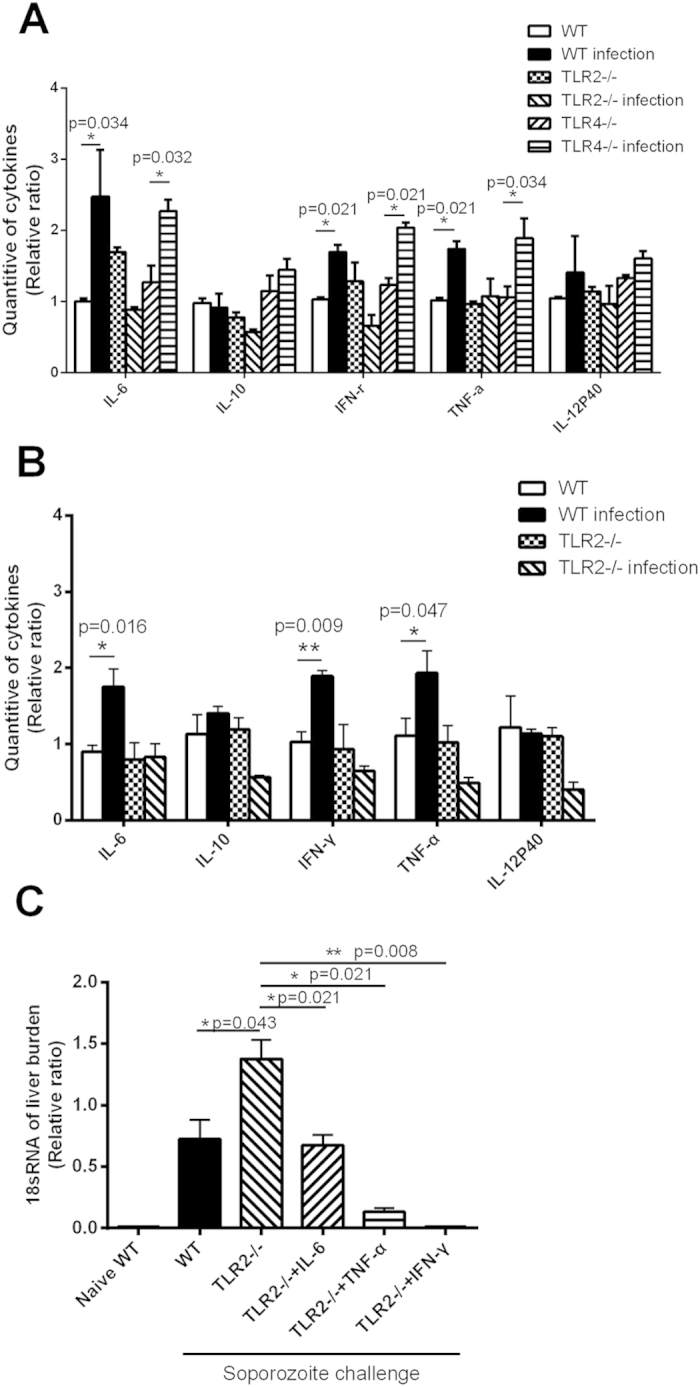
Downregulation of proinflammatory cytokine mRNA is closely associated with higher level of parasite load in TLR2^−/−^ mice. WT, TLR4^−/−^ and TLR2^−/−^ mice (n = 5) were left uninfected or challenged with sporozoites by i.v. (**A**), 42 h later, the mRNA levels of the indicated proinflammatory cytokines were determined with SYBR semi-quantitative RT-PCR. All data were presented as the ratio of the proinflammatory cytokines to the GAPDH control. (**B**), WT and TLR2^−/−^ mice (n = 5) were challenged with or without sporozoites by mosquito bite, and the proinflammatory cytokine mRNAs were determined as above. (**C**), TLR2^−/−^ mice (n = 5) challenged i.v. with sporozoites were left untreated or co-treated with IL-6, TNF-α or IFN-γ. 42 h later, the liver parasite burden was determined by real time PCR as described previously. All the experiments were repeated for three times, and all data were presented as the mean ± SD, *p < 0.05; **p < 0.01.

Previous studies have shown the critical role of proinflammatory cytokines, such as IL-12, IL-1, IL-6, TNF-α and IFN-γ, in the resistance to sporozoite challenge^29^,^30^,^31^,^32^. Therefore, we then investigated whether proinflammatory cytokines in the liver were also responsible for the inhibitory role of TLR2 activity on malaria liver-stage development. The levels of most cytokines in TLR2^−/−^ and TLR4^−/−^ mice were comparable to those in naive WT mice, indicating that the TLR2^−/−^ or TLR4^−/−^ mice have no intrinsic defect in the production of cytokines. After intravenous challenge with sporozoites, most proinflammatory cytokines, including IL-6 and TNF-α, were significantly upregulated in WT or TLR4-deficient mice (p < *0.05*), but no cytokines were elevated in the TLR2-deficient mice (Fig. 5A). Similarly, after sporozoite challenge by the infected mosquito bite, the mRNA levels of IL-6, TNF-α and IFN-γ were significantly elevated in WT mice (*p* < *0.05*), but no cytokines were upregulated in TLR2^−/−^ mice (Fig. 5B).

To confirm whether the higher level of liver parasite burden in TLR2-deficient mice was a result of the low production of proinflammatory cytokines, TLR2^−/−^ mice challenged by i.v. with sporozoites were treated with IL-6, TNF-α or IFN-γ at the same time. As shown in Fig. 5C, co-administration of sporozoites with IFN-γ could completely inhibit the development of the liver stage in the TLR2-deficient mice. Compared to TNF-α and IFN-γ, IL-6 seems to be the weakest inhibitor, but it could also reduce the parasite burden of TLR2-deficient mice to the level of naive WT mice. These data suggest that the observed high level of liver parasite load in TLR2^−/−^ mice was closely associated with reduced proinflammatory cytokine levels in the liver.

## Discussion

Recognition of invading sporozoites is the first step in initiating innate immune responses against sporozoite challenge. However, it is still unknown which TLR is involved in the recognition of sporozoites. Here, our reporter gene and macrophage-activation assays both demonstrated that sporozoites could activate TLR2 but not other tested TLRs. Interestingly, TLR2 signaling was found to be able to significantly attenuate sporozoite infection after challenged by either i.v. or infected-mosquito bite. Furthermore, we also provided evidence to show that the observed higher liver parasite load in TLR2^−/−^ mice was greatly associated with the reduced proinflammatory cytokines in the liver following challenge.

We found that whole sporozoite and its lysate induce macrophages to release proinflammatory cytokines in a TLR2^_^dependent manner (Fig. S3 & Fig. 3), and the liver parasite burden of TLR2^−/−^ mice was comparable to that of MyD88^−/−^ mice following sporozoite challenge (Fig. 4A). This could exclude the involvement of other MyD88-mediated TLRs, such as TLR5, TLR7, TLR9, TLR11 and TLR12, in this process. Although we also found that sporozoite lysate could slightly activate TLR4, it was found to be resulted from endotoxin contamination in SPZ lysate during preparation (Fig. 1C). Therefore, our data strongly support that sporozoites mainly activate TLR2 but not other tested TLRs.

Studies on the blood stage of the malaria parasite^15^, *Leishmania*^33^, *Toxoplasma gondii*^34^ and *Trypanosome*^35^ have shown that GPIs were the common TLR2 agonist of protozoa. We found that sporozoite lysates could activate either TLR2 homodimers or TLR2 heterodimers with TLR1 or TLR6 (Fig. 2A), which was consistent with the activation of TLR2/6 and TLR2/1 by GPIs from *Trypanosome cruzi*^33^ and the blood stage of malaria parasites, respectively^15^. Therefore, it is possible that the TLR2-stimulating activity of SPZ lysate might be resulted from their GPIs. In addition, we found that the capacity of whole sporozoite and its lysate to activate both TLR2 homodimers and heterodimers was greatly reduced when it was heat-inactivated or proteolyzed by proteinase K (Fig. S2 & Fig. 2B,C), indicating that the TLR2 agonist present in SPZ and its lysate might be an unknown protein. Previous and recent studies have reported that proteins, such as heat shock proteins, *Pneumococcal Pilus* RrgA^28^ and *Trypanosoma cruzi* Tc52-Released Protein^27^, could serve as TLR2 agonists. As the sporozoites were almost entirely covered by the GPI-anchored CSP (circumsporozoite protein)^36^, the role of CSP itself and its GPIs in acting as a TLR2 agonist could not be excluded.

Interestingly, we found that TLR2 could significantly attenuate *P. yoelii* infection after sporozoite challenge via either i.v. or mosquito bite (Fig. 4A,B). This result was further confirmed by the finding that co-administration of TLR2/2 agonist LTA, TLR2/1 agonist Pam3CSK4 or TLR2/6 agonist FSL could remarkably reduce the liver parasite burden of mice following intravenous sporozoite challenge (*p < 0.05*, Supplementary Fig. S4). Although we found that the higher parasite liver load in TLR2-deficient mice might be closely associated with reduced proinflammatory cytokines, such as TNF-α, IFN-γ and IL-6, in the liver following challenge (Fig. 5), the underlying mechanism was not revealed in this study.

Kupffer cells were regarded as the portal for sporozoites to enter the liver^37^,^38^; our previous studies suggested that pre-administration of individual TLR agonists could inhibit the liver stage at various levels, possibly through modulating the function of Kupffer cells^20^,^21^. It is, therefore, possible for sporozoites to activate TLR2 on Kupffer cells, and induce the cells to release proinflammatory cytokines, such as IL-1, IL-6 and TNF-α. Either TNF-α or IL-1 secreted by the activated Kupffer cells, will trigger the non-parenchymal cells to release IL-6, which was demonstrated to kill the intrahepatic parasite partially dependent on L-arginine^31^,^39^,^40^,^41^. Also, the phagocytosis capacity of the activated Kupffer cells might be enhanced and more sporozoites with less activity will be killed.

In addition to Kupffer cells, γδT^42^, NK and NKT (including Vα14 NKT^43^, DN NKT^6^ and CD1-dependent NKT^7^,^44^) in the liver were all found to be able to suppress intrahepatic parasites mainly through IFN-γ secretion. TLR2 is expressed on the surface of all of the above immune cells, therefore, it might be possible for sporozoites to directly act on those cells, and induce them to release IFN-γ. However, whether NK cells could be directly activated by TLR agonist or not is still controversial^45^,^46^. Evidence has shown that NK cells could be indirectly activated by the cytokine IL-18 released by the TLR agonist-activated Kupffer cells 47. Therefore, it is also possible for the TLR2 agonist present in sporozoites to indirectly trigger NK cells to release IFN-γ through the cross-talking between NK cells and Kupffer cells.

In conclusion, to our knowledge, we provided the first evidence to show that sporozoites contain a TLR2 agonist that can elicit an innate immune response to effectively resist sporozoite challenge. Our data could not only help us to understand the mechanism of innate immune responses against sporozoite challenge but also provide us with novel clues for designing preventive therapies against the pre-erythrocytic stage of malaria.

## Materials and Methods

### Mice and plasmodium

TLR2^−/−^,TLR4^−/−^, and MyD88^−/−^mice (C57BL/6J background) were from The Jackson Laboratory (Bar Harbor, ME). Specific pathogen-free wild-type (WT) C57BL/6J mice were purchased from the Model Animal Research Center of Nanjing University. Kunming mice (a kind of Swiss Webster mice) were from the Experimental Animal Center of the Third Military Medical University. *P. yoelii* (*Plasmodium yoelii*) *yoelii* 265BY, the uncloned parasite, is isolated in 1969 in Center Africa Republic^48^, and maintained by passage between Kunming mouse and mosquito. All methods were carried out in accordance with the approved Guide for the Care and Use of Laboratory Animals of the Third Military Medical University. All experimental protocols were approved by the Animal Institute of Third Military Medical University.

### Mosquito rearing and infection

*Anopheles stephensi* (Hor strain) were maintained at 27 °C, 70–80% relative humidity and fed with 5% sugar solution. For infection with *P. yoelii*, 3- to 5-day old female adults were kept at 23–24 °C and fed on *P. yoelii yoelii* 265BY-infected Kunming mice, when the gametocytemia of the infected mice was ≥0.5%. At 7 days following infection, mosquitoes were dissected, and the oocysts on the midguts were examined under a light microscope.

### Sporozoite purification and sporozoite lysate preparation

At 17 days following infection with *P.yoelii yoelii* 265BY, ~300 infected- or uninfected-female mosquitoes were anesthetized and washed in 75% ethanol to remove surface contamination, respectively. After the mosquitoes were extensively washed in the sterile PBS, the salivary glands were dissected and collected in RPMI 1640 containing 2.5 μg/ml amphotericin B (Sangon Biotech, China),100 units/ml penicillin and 100 μg/ml streptomycin (Beyotime, China). Next, the salivary glands from the infected- or uninfected mosquitoes were ground in a 1.5 ml tube using a plastic grinding bar, and the released sporozoites were purified by DEAE cellulose chromatography as previously reported 49. Both sporozoite lysate and normal salivary gland lysate were prepared by repeated freezing and thawing of the purified sporozoites for four cycles. The whole sporozoites lack of infectivity were prepared by placing in medium at 4 °C for more than 24 h as previously described^24^,^25^.

### Detection of the endotoxin and bacterial content of sporozoite lysate

The endotoxin level in the sporozoite lysate and normal salivary gland lysate were detected using Limulus Amebocyte Lysate test kit E-TOXATE®,(Sigma, USA). To quantify bacterial contamination, serial dilutions of SPZ (sporozoite) lysate and normal salivary gland lysate were plated onto LB (Luria Bertani) medium agar plates and incubated at 37 °C for 16 h.

### Intravenous or mosquito bite challenge with sporozoites

At 17 days following infection, sporozoites were isolated from the infected mosquitoes as described as above. WT, TLR2^−/−^, TLR4^−/−^ and MyD88^−/−^ mice were challenged by i.v. with 250 sporozoites, respectively. Alternatively, mice were bitten for 30 minutes by a pot of 20 mosquitoes that had been infected with *P. yoelii yoelii* 265BY for 17 days. To test the influence of pro-inflammatory cytokines on liver-stage development, TLR2^−/−^ mice were challenged with sporozoites following intravenously injection of 5 μg recombinated mouse IFN-γ (Peprotech), TNF-α (Peprotech) or IL-6 (Peprotech), respectively. The parasite load in the liver was determined by TaqMan real-time PCR.

### Mice infected with blood stage malaria

Each WT and TLR2^−/−^ mouse was injected intraperitoneally with 1 × 10^6^ *P.y yoelii* 265BY-infected RBC. The parasitemia in the blood was determined by Giemsa staining every two days.

### Construction of recombinant mouse TLR plasmids

Total RNA from the livers or spleens of C57BL/6J mice was isolated with Trizol (Invitrogen, USA) and reverse transcribed. The obtained cDNA was used as a template to amplify (KOD FX DNA polymerase) the coding sequences of TLR1, TLR3, TLR5, TLR7, TLR9 and CD14. Next, the target fragments were cloned into pcDNA3.1, and the inserts were verified by sequencing. Mouse TLR2, MD-2 and TLR4 were amplified from pFLAG-cmv8-mTLR2 (a gift from Dr. Xin Du, Scripps Research Institute), pEFBOS-MD2 and pEFBOS-TLR4 (gifts from Dr. Kensuke Miyake, University of Tokyo), and subcloned into pcDNA3.1, respectively.

### Dual-luciferase assays for NF-κB and IFN-β gene reporter activities detection

HEK293FT cells were cultured in RPMI 1640 (Hyclone, USA) supplemented with 10% heat-inactivated FBS (fetal bovine serum) at 37 °C under humidified air containing 5% CO_2_. 3 × 10^4^ HEK 293FT cells/well were plated in 96-well plates. Each well was transfected with Lipofectamine 2000 (Invitrogen, USA), 2 ng TK-RL (Promega, USA) and 200 ng of an individual TLR (TLR3, TLR5, TLR7 or TLR9) or 100 ng each TLR4 and MD-2, or 100 ng each TLR2 and CD14, along with 200 ng pBIIx-luc reporter gene (a gift from Dr. Sankar Ghosh’s Lab, Yale University) or pGL3-IFN-β (gift from Dr. Rongtuan Lin’s Lab, McGill University). Twenty-four hours later, cells were stimulated with the corresponding TLR agonist, such as 1 μg/ml LTA-BS(Lipoteichoic Acid from *Bacillus subtilis*, Invivogen, USA), 100 ng/ml LPS(Invivogen, USA), 2.5 μg/ml Flagellin(Invivogen, USA), 25 μg/ml PolyI:C(Invivogen, USA), 3 μg/ml Gardiquimod^TM^(Invivogen, USA), or 10 μg/ml CpG ODN (Invivogen, USA), equivalent amounts of SPZ lysate or NSG lysate. After incubation for 6 h, TLR2/CD14-, TLR4/MD-2-, TLR5-, TLR7- or TLR9-transfected cells were lysed, and NF-κB gene reporter activity was determined; TLR3-transfected cells were assessed for IFN-β gene reporter activity using Dual-luciferase assay kit (Promega, USA) after stimulation for 18 h.

To investigate whether SPZ lysate could activate TLR2 homodimer and/or heterodimer, cells were transfected with 100 ng CD14 in combination with 100 ng TLR2, 50 ng TLR2 and 50 ng TLR1 or 50 ng TLR2 and 50 ng TLR6 and then stimulated with their corresponding agonists (1 μg/ml LTA, 100 ng/ml Pam3CSK4 or 100 ng/ml FSL-1), SPZ lysate or NSG lysate. To investigate whether the proteins in SPZ lysate activate the TLR2, TLR2/2-, TLR2/1-, and TLR2/6-transfected cells were stimulated with unheated or heated (boiling water bath for 20 min) SPZ lysate. Additionally, SPZ lysate or NSG lysate was proteolyzed by incubation with 50 ug/ml proteinase K agarose (Sigma) at 37 °Cfor 3 h, then the supernatant was collected after centrifugation. TLR2-transfected cells were then stimulated with SPZ lysate or NSG lysate pre-treated with or without proteinase K. After incubation for 6 h, cells were lysed and NF-κB gene reporter activity was determined as above.

### Isolation of peritoneal macrophages and cytokine measurement

After WT or TLR2^−/−^ mice were intraperitoneally injected with 1 ml 3% TGC (Thioglycollate, Sigma, USA) for three days, peritoneal macrophages were extracted and allowed to adhere on tissue culture dishes for two hours, and non-adherent cells were removed. Next, adherent cells were collected, and 5 × 10^4^ cells were plated in 96-well plates and left at 37 °C overnight. To test the LPS contaminating in SPZ lysate, peritoneal macrophages from the WT mice were then stimulated with 100 ng/ml LPS, 1 × 10^5^ SPZ lysate or equivalent amounts of NSG lysate treated with or without 2.5 μg/ml PmB (polymyxin B, Sigma) for 22 h. To investigate whether SPZ lysate could activate the macrophage in a TLR2-dependent manner, macrophages collected from WT, TLR2^−/−^ and TLR4^−/−^ mice were stimulated with 2 × 10^5^ SPZ lysate or equivalent amounts of NSG lysate or FSL-1(invivogen) or LPS (invivogen) for 17 h. Supernatant was then collected, and the concentration of TNF-a, IL-6 and MCP-1 was determined by FACS flow cytometry using the CBA mouse inflammation kit (BD, USA) according to the manufacturer’s protocol.

### TaqMan real-time PCR assay for liver parasite burden

Parasite load in the liver was determined by detection of *plasmodium*-specific 18S rRNA using real-time PCR as previously described^20^. In brief, at 42 h following sporozoite challenge, livers from WT, TLR2^−/−^, TLR4^−/−^ and MyD88^−/−^ mice were isolated, and total RNA was isolated by Trizol (Invitrogen, USA) and reverse transcribed using random primers. Next, real-time PCR reactions for 18S rRNA and GAPDH were established using Premix Ex Taq (GeneCore BioTechnologies Co, Vita Genomics, Inc). Real-time PCR was performed using the Eco qPCR system (Illumina, San Diego, USA, Inc), and the parasite burden of each unknown sample was expressed as the ratio of the parasite 18S rRNA to mouse GAPDH.

### SYBR quantitative PCR assay for liver cytokine mRNA detection

SYBR quantitative analysis of mRNA expression was used to investigate the expression of cytokines, including IL-6, IL-12, IL-10, TNF-α and IFN-γ, in the sporozoite-challenged WT, TLR2^−/−^, TLR4^−/−^ mice liver. PCR reactions (15 μl) contained 0.25 μl of each primer (10 μM), 7.5 μl SYBR^®^ Premix Ex Taq TM II 2 × (TAKARA, Japan), 6 μl ddH_2_O and 1 μl cDNA. The PCR conditions consisted of an initial denaturation at 95 °C for 30 s followed by amplification for 40 cycles of 15 s at 95 °C and 50 s at 60 °C, with fluorescence acquisition at the end of each extension step. PCR using primers for GAPDH was performed on each individual sample as an internal control. The optical density of each PCR band was measured semi-quantitatively using Illumina Eco software (Illumina, San Diego, USA, In).

### Statistical analysis

All the data were analyzed by Graphad Prism 5.0 version. Mann Whitney test was used to compare two groups, and data more than two groups was analyzed with Kruskal-Willis test, and Log Rank test was used for survival rate analysis. *P < 0.05* was considered to be statistically significant.

## Additional Information

**How to cite this article**: Zheng, H. *et al.* The TLR2 is activated by sporozoites and suppresses intrahepatic rodent malaria parasite development. *Sci. Rep.* **5**, 18239; doi: 10.1038/srep18239 (2015).

## Supplementary Material

Supplementary Information
