# Supplementary material for: The TLR2 is activated by sporozoites and suppresses intrahepatic rodent malaria parasite development
Source: Sci Rep. 2015 Dec 15;5:18239. doi: 10.1038/srep18239 (PMC4678895; doi:10.1038/srep18239)
Supplement: Supplementary Information [file srep18239-s1.doc]

**The TLR2 signaling pathway is activated by sporozoites and suppresses intrahepatic rodent malaria parasite development**

Hong Zheng, Zhangping Tan, TaoLi Zhou, Feng Zhu, Yan Ding, Taiping Liu, Yuzhang Wu, Wenyue Xu

**Methods**

***Preparing of the purified whole sporozoites***

According to the method reported in 1978[^1^](#_ENREF_1), salivary glands with sporozoites were dissected from 17-day infected female *Anopheles stephensi* and collected into a 1.5ml EP tube with 500ul RPMI 1640(Penicillin, Streptomycin, Amphotericin B, Gentamicin). Then salivary glands were collected by centrifugation(16000g, 10min) at 4℃ and supernatant liquid were discarded. Using 1ml filtered column buffer to resuspend the precipitation and salivary glands were completely undermined by using 1ml syringe. Then the suspension were purified by through DE52 column, and the purified sporozoites were collected in 40ml [liquid](javascript:void(0);) flowed from the column in an ice bath. The whole sporozoites were collected by centrifugation(16000g, 10min) at 4℃ and supernatant liquid were discarded. The sporozoites were counted and adjusted to 6x10^4^ sporozoites/5 ul buffer. To inactivate the parasite, the whole sporozoites were placed in refrigerator at 4℃ for 24h. The normal salivary glands(NSG) was prepared in the same way, and the amount of mosquitoes in NSG groups was equal to whole sporozoites group.

***Preparing of whole sporozoites stimulant***

The inactivated whole sporozoites were then treated by 20 min-boiling in water bath, and an equal volume of NSG were prepared in the same way. Additionally, whole sporozoites or NSG was proteolyzed by incubation with 50ug/ml proteinase K agarose (Sigma) at 37℃ for 3h[^2^](#_ENREF_2), the control groups(whole SPZ+buffer and NSG+buffer) were incubated at 37℃ for 3h without proteinase K agarose at the same time.

***The effect of activation of TLR2 signaling on the development of pre-erythrocytic stage*** WT mice pretreated with or without 20 μg TLR2/2 agonist LTA-BS (invivogen), TLR2/1 agonist Pam3CSK4 (invivogen) or TLR2/6 agonist FSL-1 (invivogen), were then challenged with 250 sporozoites by i.v.

**Figure S1**

Figure S1.
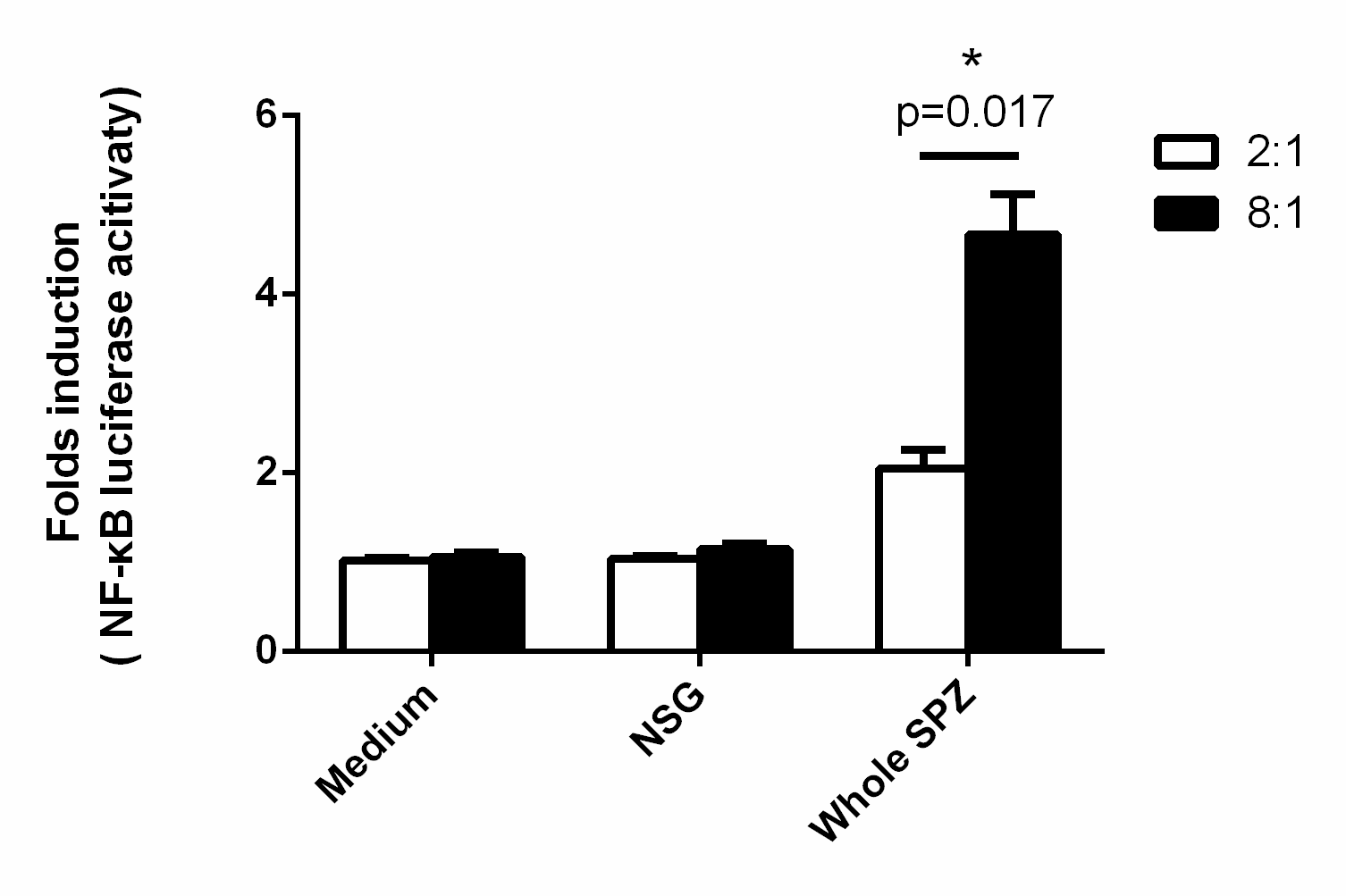
 Dose-dependent of whole sporozoites activity. 3×10^4^ TLR2/2-transfected HEK 293FT cells were stimulated with 6×10^4^(5ul) whole sporozoites and NSG(5ul) or 2.4×10^5^(20ul) whole sporozoites and NSG(20ul) for 6h. Then the NF-κB gene reporter activity was then determined. The experiment was repeated three times, and all data were presented as the mean ± SD, * p<0.05.

**Figure S2**

**
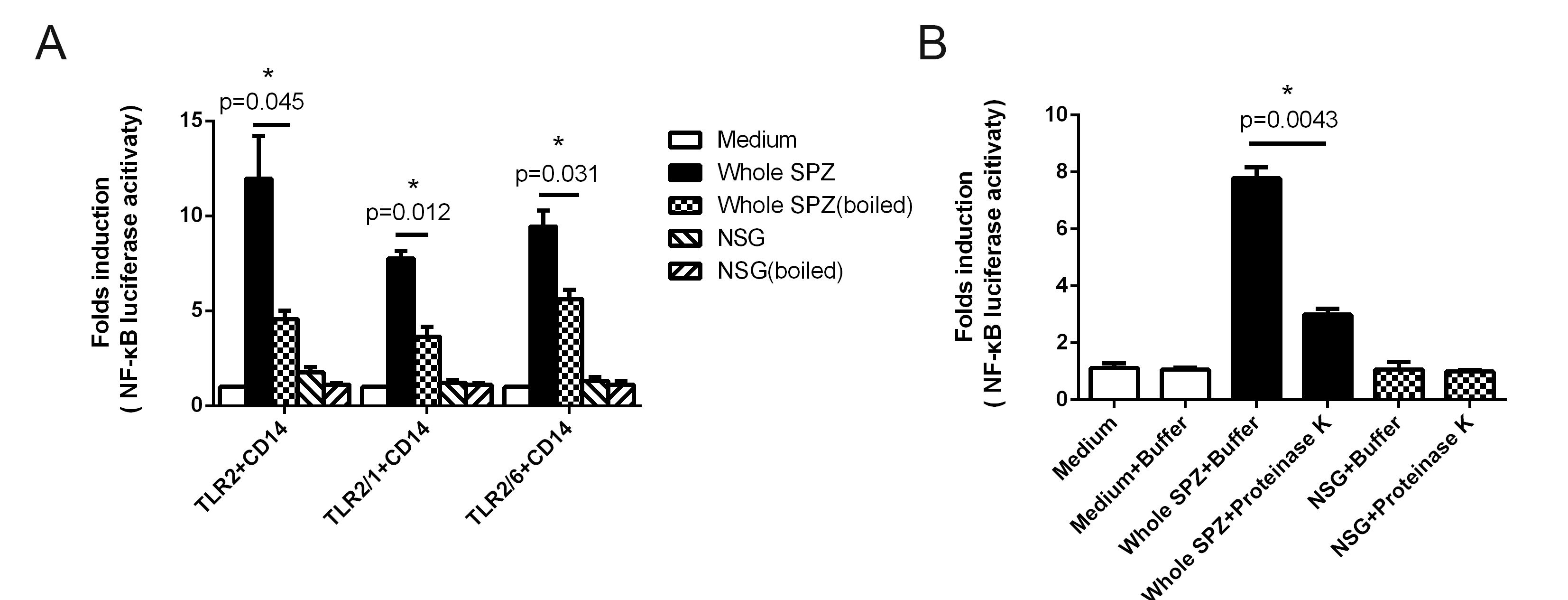
**

Figure S2. Characterization of TLR2 agonist contained in whole sporozoites. A, TLR2/CD14-, TLR2/1/CD14- or TLR2/6/CD14-transfected cells were stimulated with boiled or unboiled whole SPZ or NSG, and then NF-κB gene reporter activity was determined. B, TLR2/2/CD14 -transfected cells (cell/sporozoites=1:8) were stimulated with the whole SPZ or NSG pretreated with or without protease K, and the NF-κB gene reporter activity was then determined. All the experiments were repeated three times, and all data were presented as the mean ± SD, * p<0.05.


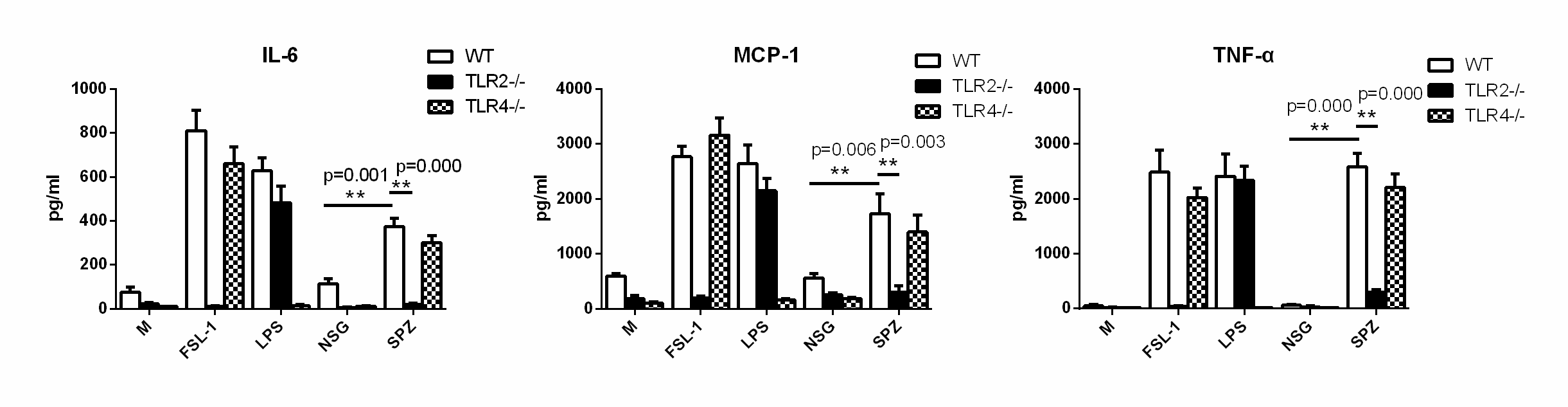
**Figure S3**

Figure S3. Whole sporozoites induced peritoneal macrophages to release proinflammatory cytokines in a TLR2-dependent manner. Peritoneal macrophages collected from WT, TLR2^-/-^ or TLR4^-/-^ mice were stimulated with or without whole SPZ, FSL-1, LPS or NSG. 17 h later, the concentration of IL-6, MCP-1 and TNF-α in the supernatant was determined using CAB beads. The experiment was repeated three times, and all data were presented as the mean ± SD, * *p<0.05.*


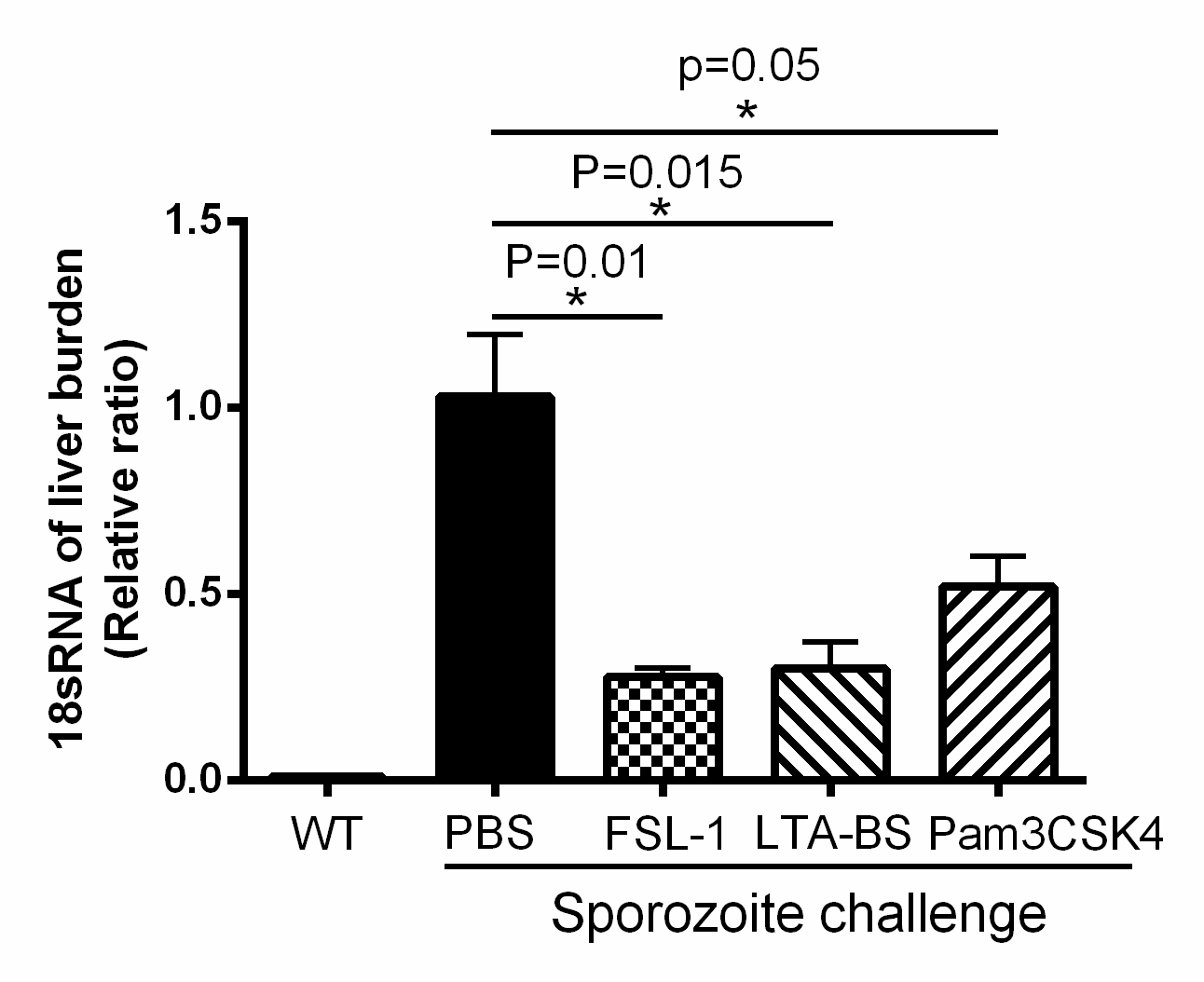
**Figure S4**

Figure S4. Effects of TLR2/2, TLR2/1 and TLR2/6 agonists on the development of the pre-erythrocytic stage. WT mice (n=5) treated with TLR2/2 agonist LTA-BS, TLR2/1 agonist Pam3CSK4 or TLR2/6 agonist FSL-1 were then challenged intravenously with sporozoites. 42 h later, liver parasite burden was detected by quantitative PCR. The experiment was repeated for three time, and all the data were presented as the mean ± SD, * *p<0.05*

1 Mack, S. R., Vanderberg, J. P. & Nawrot, R. Column separation of Plasmodium berghei sporozoites. *J. Parasitol.* **64**, 166-168 (1978).

2 Luong, M. *et al.* Stimulation of TLR4 by recombinant HSP70 requires structural integrity of the HSP70 protein itself. *J Inflamm (Lond)* **9**, 11 (2012).
